# Engineering Nanotherapeutics Through Interfacial Mechanics: Mechanobiology and Precision Drug Delivery

**DOI:** 10.3390/mi17091086

**Published:** 2026-09-16

**Authors:** Alireza Mohammad Karim

**Affiliations:** Ingram School of Engineering, Texas State University, San Marcos, TX 78666, USA; alireza.m.karim@txstate.edu

**Keywords:** mechanobiology, nanotherapeutics, interfacial mechanics, mechanotransduction, precision drug delivery

## Abstract

The biological microenvironment is inherently mechanical, with cells continuously sensing and responding to fluid shear stress, matrix stiffness, tensile strain, hydrostatic pressure, and other physical cues through highly conserved mechanotransduction pathways. These mechanical interactions arise across biological interfaces and are increasingly investigated using microfluidic and nanofluidic technologies that enable precise control of physiologically relevant mechanical environments. While nanomedicine has traditionally focused on biochemical targeting and molecular recognition, growing evidence demonstrates that interfacial mechanical forces critically regulate nanoparticle transport, vascular adhesion, cellular uptake, biodistribution, therapeutic activation, and treatment efficacy. Simultaneously, pathological alterations in tissue mechanics have emerged as hallmarks of numerous diseases, including cancer, cardiovascular disease, fibrosis, pulmonary hypertension, neurodegeneration, and metabolic disorders, creating new opportunities for mechanics-guided therapeutic design. This Perspective develops an integrative framework in which interfacial mechanics is treated as an actionable engineering variable across the nanotherapeutic delivery pathway. The framework distinguishes three mechanistic roles of mechanics: as a determinant of therapeutic transport, as an activation signal for mechanically responsive materials, and as a biological target through mechanotransduction pathways. Particular attention is given to fluid shear stress, extracellular-matrix mechanics, strain, pressure, nano–cell interfacial interactions, and the use of microfluidic and nanofluidic systems to reproduce physiologically relevant mechanical environments. Experimentally demonstrated mechanically responsive systems are distinguished from prospective concepts, and the translational implications of mechanical heterogeneity, activation thresholds, off-target triggering, and combined mechanical–biochemical gating are examined. The Perspective further identifies quantitative and experimental requirements needed to advance mechanics-guided nanotherapeutics from conceptual designs toward predictive and clinically translatable systems. Collectively, this framework positions interfacial mechanics as a complementary design dimension to molecular targeting in precision drug delivery.

## 1. Introduction

Over the past three decades, nanomedicine has transformed the landscape of therapeutic delivery by enabling unprecedented control over drug transport, biodistribution, pharmacokinetics, and site-specific targeting [1,2]. Advances in nanoparticle engineering, biomaterials design, and molecular targeting strategies have generated a diverse arsenal of nanotherapeutic platforms, including liposomes, polymeric nanoparticles, dendrimers, inorganic nanomaterials, extracellular vesicles, and self-assembled supramolecular systems [2,3]. These technologies have significantly expanded the therapeutic window of numerous drugs while reducing systemic toxicity and improving treatment efficacy across a broad spectrum of diseases, including cancer, cardiovascular disorders, infectious diseases, and metabolic syndromes [1,4,5].

Disease-associated mechanics can provide information that is complementary to biochemical and molecular biomarkers. Fluid shear stress, extracellular-matrix stiffness, tissue deformation, pressure, and related physical variables influence therapeutic transport while also reflecting pathological remodeling. These mechanical features can therefore be considered not only as constraints that a nanotherapeutic must overcome, but also as measurable variables that may inform carrier design, activation, localization, and therapeutic intervention.

The conceptual foundation of modern nanomedicine has been largely built upon chemical and molecular principles [6,7]. Considerable effort has been devoted to engineering particle composition, surface chemistry, targeting ligands, receptor specificity, molecular recognition, and stimuli-responsive release mechanisms [7,8]. Consequently, the design of most contemporary nanotherapeutics is guided primarily by biochemical considerations, including receptor expression profiles, enzymatic activity, pH gradients, redox environments, inflammatory mediators, and disease-specific molecular biomarkers [2,8]. These approaches have yielded substantial progress and remain essential components of precision medicine. Despite these advances, an important aspect of the biological environment remains comparatively underexplored in nanotherapeutic design: mechanics.

Recent advances in microfluidic, nanofluidic, and organ-on-chip technologies have transformed our ability to investigate the mechanical regulation of therapeutic transport within physiologically relevant environments. By enabling precise control of fluid flow, shear stress, pressure gradients, endothelial interfaces, extracellular matrix (ECM) architecture, and microscale transport, these platforms provide powerful experimental systems for reproducing complex in vivo mechanical microenvironments. Beyond serving as in vitro disease models, microfluidic technologies increasingly function as engineering platforms for understanding mechanobiology, evaluating nanotherapeutic performance, and accelerating the development of mechanically responsive drug-delivery systems. Consequently, the convergence of interfacial physics, microfluidics, mechanobiology, and nanomedicine is establishing a new interdisciplinary framework for precision therapeutic engineering.

Every stage of therapeutic delivery occurs within a mechanically active environment [9,10]. Following administration, nanotherapeutics encounter complex hemodynamic forces generated by blood flow [9,11,12]. Their transport through the circulation is governed by fluid shear stresses, pressure gradients, and hydrodynamic interactions with blood cells and vascular walls [9,11,12]. Subsequent vascular adhesion, extravasation, and penetration into diseased tissues are influenced by vessel mechanics, endothelial permeability, interstitial pressure, ECM architecture, and tissue stiffness [13,14,15]. At the cellular level, nanoparticle internalization is regulated by membrane tension, cytoskeletal dynamics, and force-dependent endocytic pathways, while intracellular trafficking is strongly influenced by molecular motors and mechanically organized cytoskeletal networks [16,17,18,19,20]. Consequently, mechanical forces influence virtually every step of the drug delivery cascade, from systemic administration to therapeutic response.

Parallel advances in mechanobiology have fundamentally changed our understanding of how cells interact with their physical environment [21,22]. It is now well established that cells continuously sense, interpret, and respond to mechanical signals arising from ECM stiffness, fluid flow, tensile strain, compressive stress, and hydrostatic pressure [23,24]. Through specialized mechanotransduction machinery, including integrins, focal adhesions, cadherins, primary cilia, cytoskeletal networks, and mechanosensitive ion channels such as PIEZO1 and PIEZO2, physical stimuli are converted into biochemical signals that regulate cellular behavior, gene expression, metabolism, proliferation, differentiation, and survival [24,25,26,27,28]. These discoveries have revealed that mechanics is not merely a passive consequence of biological activity but rather a fundamental regulator of cellular function [25,29].

Importantly, pathological progression is frequently accompanied by profound alterations in tissue mechanics. Increased matrix stiffness is a hallmark of fibrosis and solid tumors [29,30,31,32]. Disturbed flow patterns and abnormal shear stress contribute to atherosclerosis and vascular remodeling [33,34]. Altered cyclic strain regulates cardiac hypertrophy and heart failure progression [35,36]. Elevated interstitial pressure influences tumor transport barriers, while abnormal mechanical loading contributes to degenerative musculoskeletal diseases [14,15,25,36]. Such observations suggest that mechanical abnormalities are not simply byproducts of disease but active participants in pathological development and therapeutic outcomes.

Existing nanomedicine literature has extensively addressed physicochemical carrier design, biological barriers, particle transport, tumor microenvironmental constraints, and cellular mechanotransduction. However, these subjects are commonly treated as separate domains. Reviews of precision nanoparticles have primarily emphasized particle composition, geometry, surface chemistry, targeting, and biological barriers, whereas work on tumor mechanics has largely focused on how solid stress, stiffness, and interstitial pressure impede therapeutic transport. Mechanobiology literature, in turn, has generally examined how cells sense and respond to force rather than how mechanical information can be incorporated systematically into nanotherapeutic design.

The distinctive contribution of this Perspective is therefore an integrated engineering framework that connects four levels that have largely remained fragmented: disease-associated mechanical properties and loading conditions; mechanics-dependent transport and nano–bio interfacial interactions; mechanically responsive therapeutic materials; and mechanotransduction pathways that may themselves be therapeutic targets. Within this framework, mechanics is separated into three functional roles: a transport determinant that governs carrier motion and biological access, an activation signal that can trigger material or drug response, and a therapeutic target whose cellular interpretation can be pharmacologically modulated. This distinction provides a practical design logic for deciding where and how mechanical information can be incorporated into nanotherapeutic development.

**Figure 1:**
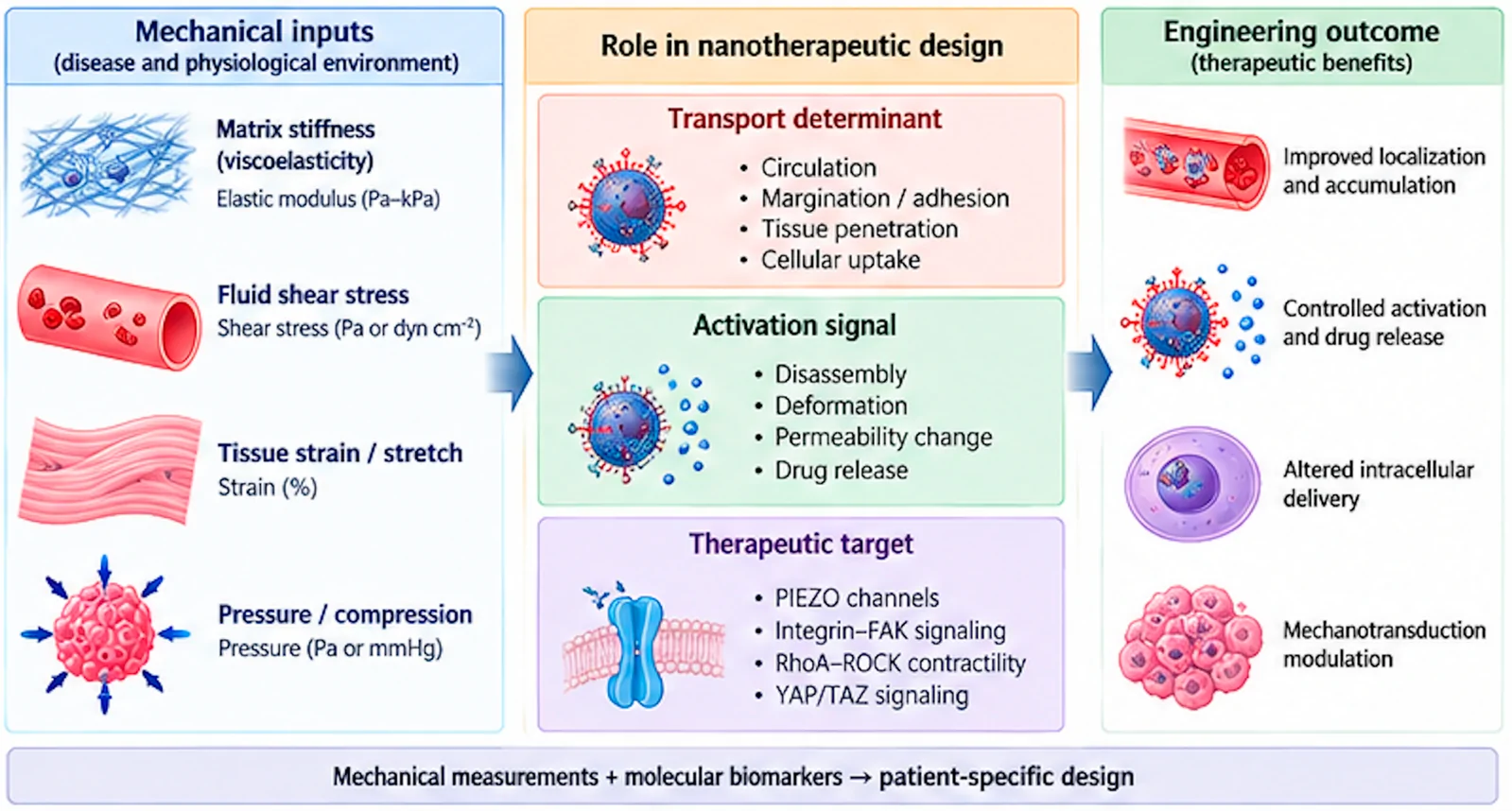
Interfacial mechanics as a design framework for nanotherapeutics. Biological mechanics can influence nanotherapeutic performance through three distinct roles: as a transport determinant governing carrier motion and biological access; as an activation signal capable of inducing material reconfiguration or therapeutic release; and as a biological target through mechanotransduction pathways. The framework distinguishes these roles to connect measurable mechanical properties and loading conditions with specific engineering outcomes. Mechanical information is proposed as complementary to, rather than a replacement for, molecular targeting.

The Perspective consequently examines the mechanical properties and loading conditions encountered in biological systems, their influence across the nanotherapeutic delivery cascade, experimentally demonstrated and emerging mechanically responsive systems, nano–cell interfacial mechanics, supramolecular therapeutic materials, and mechanotransduction-targeted strategies. Particular emphasis is placed on quantitative activation criteria, experimental maturity, mechanical heterogeneity, translational safety, and the role of microfluidic, nanofluidic, computational, and patient-specific methods in establishing predictive design rules. Figure 1 summarizes this mechanics-guided framework.

## 2. Mechanical Properties and Loading Conditions in the Biological Microenvironment

The biological mechanical microenvironment is defined by both intrinsic material properties and externally or internally generated loading conditions [22,35]. These categories should be distinguished because they describe different physical quantities and have different implications for nanotherapeutic design. Extracellular-matrix stiffness, viscoelasticity, porosity, and architecture characterize the mechanical state of the surrounding material, whereas fluid shear stress, tensile or compressive strain, and hydrostatic or interstitial pressure describe loading conditions imposed on cells, tissues, or therapeutic materials [24,25].

**Figure 2:**
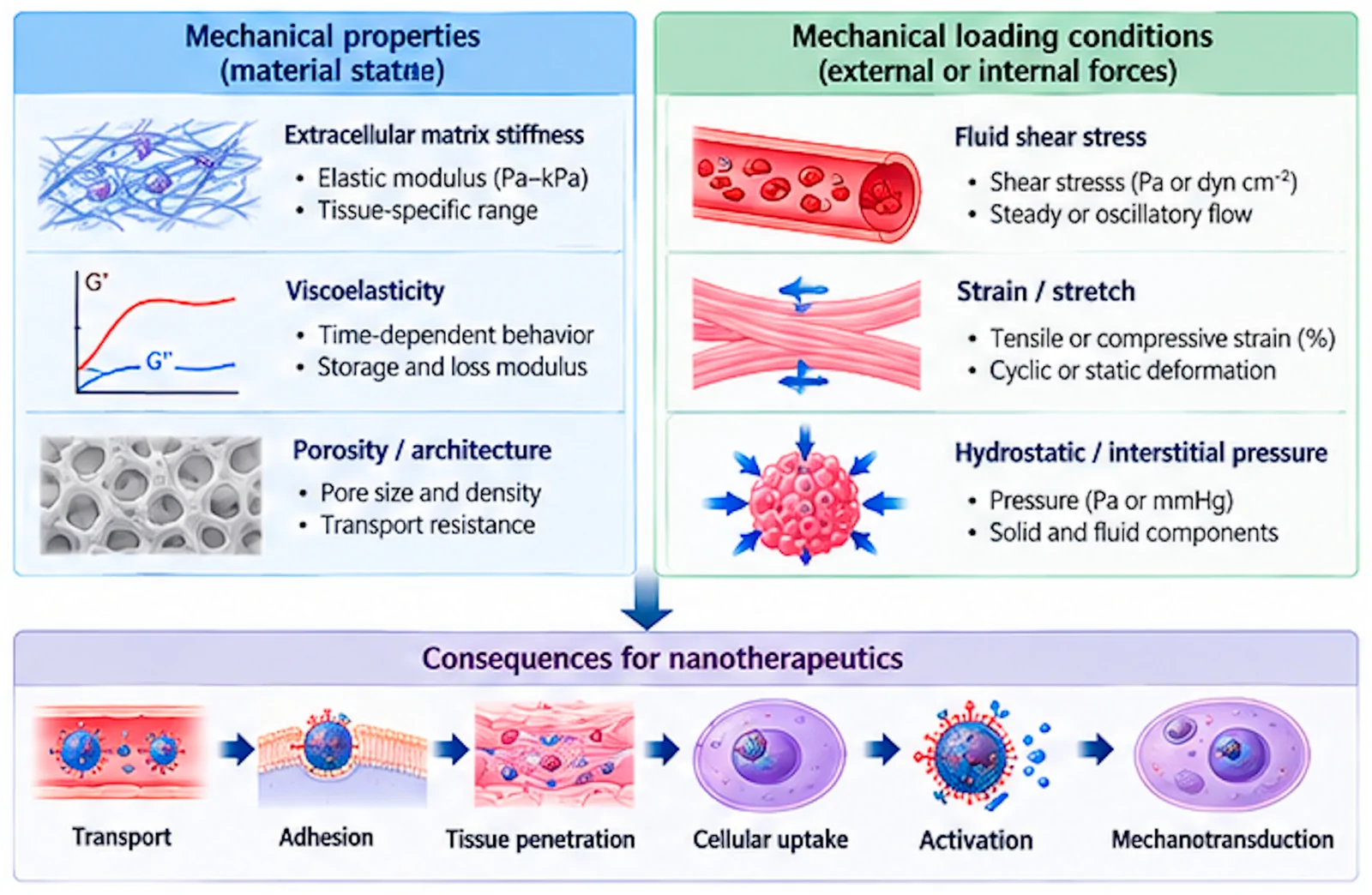
Classification of mechanical variables relevant to nanotherapeutic design. The biological mechanical microenvironment contains both material properties, such as extracellular-matrix stiffness, viscoelasticity, and porosity, and loading conditions, such as fluid shear stress, strain, and pressure. These variables influence nanotherapeutic transport, biological interaction, cellular uptake, mechanical activation, and mechanotransduction through physically distinct mechanisms.

This distinction is particularly important for mechanics-guided nanomedicine. Material properties can regulate particle penetration, cellular phenotype, membrane mechanics, and mechanotransduction continuously, whereas dynamic loading can alter carrier transport, adhesion, deformation, or trigger force-sensitive materials. Disease frequently modifies both categories simultaneously. Figure 2 therefore organizes the mechanical microenvironment into material properties and mechanical loading conditions before linking them to biological and nanotherapeutic consequences.

### 2.1. ECM Stiffness

ECM stiffness is an intrinsic mechanical property of the tissue microenvironment and one of the most extensively studied regulators of cellular behavior [23,37]. Biological tissues span broad ranges of elastic modulus, from highly compliant neural tissues to substantially stiffer muscle, cartilage, and mineralized tissues. Cells detect these differences through mechanically coupled adhesion and cytoskeletal systems, allowing local matrix mechanics to influence cell shape, migration, proliferation, differentiation, and gene expression.

Pathological remodeling frequently shifts this mechanical state. Fibrosis is characterized by excessive ECM deposition and progressive tissue stiffening [30,31], whereas many solid tumors develop increased rigidity through collagen deposition, crosslinking, stromal activation, and elevated cellular contractility [29,32]. These changes influence not only cell phenotype but also nanotherapeutic penetration and distribution by modifying matrix density, pore structure, hydraulic resistance, and cellular mechanics. Stiffness is therefore relevant to nanotherapeutics both as a transport property of diseased tissue and as a potential parameter for mechanics-informed therapeutic design.

At the cellular level, matrix stiffness is sensed through integrins and focal adhesions, which mechanically connect ECM proteins to the cytoskeleton [38,39]. Increased rigidity can promote focal-adhesion maturation, cytoskeletal tension, and nuclear localization of Yes-associated protein (YAP) and transcriptional co-activator with PDZ-binding motif (TAZ), thereby activating transcriptional programs associated with proliferation, fibrosis, and tumor progression [40,41]. These signaling pathways provide a molecular connection between the material mechanics of the extracellular environment and potential mechanotherapeutic targets.

### 2.2. Fluid Shear Stress

Fluid shear stress represents one of the most important mechanical stimuli encountered by cells within the cardiovascular system [33,42]. Generated by the frictional forces exerted by flowing fluids on cellular surfaces, shear stress plays a central role in vascular development, endothelial homeostasis, inflammation, and disease progression.

The vascular endothelium forms the primary mechanosensitive interface between circulating blood and the vessel wall. Endothelial cells continuously sense hemodynamic forces and respond through coordinated biochemical and transcriptional programs that regulate vascular tone, permeability, thrombosis, inflammation, and remodeling [34,43]. Under physiological conditions, endothelial cells are exposed to laminar shear stresses typically ranging from approximately 10 to 70 dyn∙cm^−2^ depending on vessel type and anatomical location [44]. These forces promote endothelial quiescence, anti-inflammatory signaling, nitric oxide production, and vascular homeostasis.

Laminar, disturbed, and oscillatory flow impose distinct mechanobiological programs on the vascular endothelium. Sustained laminar shear promotes endothelial alignment, nitric oxide signaling, Krüppel-like factor 2 (KLF2)/endothelial nitric oxide synthase (eNOS) activation, and anti-inflammatory vascular homeostasis, whereas disturbed or oscillatory shear at bifurcations, curvatures, and stenotic regions promotes endothelial dysfunction, oxidative stress, permeability, leukocyte recruitment, and pro-atherogenic signaling [11,12,33,34,45,46,47,48,49,50]. These hemodynamic differences are directly relevant to nanotherapeutic delivery because local flow fields regulate particle margination, endothelial encounter frequency, adhesion stability, and detachment forces.

### 2.3. Strain and Stretch

Many biological tissues experience repetitive deformation during normal physiological function. Cyclic strain and stretch therefore represent essential mechanical regulators of cellular behavior in mechanically active organs [35,36]. Dynamic organs such as the heart, lungs, and blood vessels experience continuous cyclic deformation. Cardiac contraction, respiratory expansion, and pulsatile vascular loading regulate cytoskeletal organization, nuclear mechanics, focal adhesion remodeling, ECM turnover, and gene expression. Pathological strain patterns contribute to cardiac hypertrophy, heart failure, ventilator-induced lung injury, vascular remodeling, aneurysm formation, and restenosis [35,36,51,52]. For nanotherapeutics, strain-sensitive environments may provide opportunities for delivery systems that couple therapeutic release to tissue motion or mechanical dysfunction.

### 2.4. Hydrostatic and Interstitial Pressure

Pressure represents another important component of the mechanical microenvironment. Hydrostatic and interstitial pressures influence tissue transport, vascular function, cellular signaling, and disease progression.

Solid tumors frequently exhibit elevated interstitial fluid pressure resulting from abnormal vascular architecture, impaired lymphatic drainage, and excessive ECM deposition. Increased interstitial pressure creates substantial barriers to therapeutic transport by reducing convective flow and limiting nanoparticle penetration into tumor tissues [13,14]. Consequently, elevated tumor pressure is widely recognized as a major obstacle to effective drug delivery. In addition to transport limitations, elevated pressure influences cellular behavior through mechanotransduction pathways that regulate proliferation, migration, invasion, and therapeutic resistance. Thus, pressure contributes both directly and indirectly to tumor progression [13].

Pulmonary hypertension is characterized by elevated pulmonary arterial pressure and progressive vascular remodeling. Increased pressure alters endothelial function, smooth muscle cell behavior, ECM production, and inflammatory signaling [51,53]. Persistent pressure overload drives structural changes in pulmonary vessels, ultimately increasing vascular resistance and impairing cardiac function. More broadly, pressure-mediated mechanotransduction contributes to numerous pathological processes involving vascular dysfunction, edema formation, tissue remodeling, and chronic inflammation [53].

## 3. Mechanics and Nanoparticle Transport

The therapeutic efficacy of nanomedicines is fundamentally dependent on their ability to successfully navigate a series of biological transport barriers before reaching their intended targets [2,7]. Traditionally, nanoparticle transport has been interpreted primarily through physicochemical parameters such as particle size, surface charge, hydrophobicity, and ligand density [11]. While these factors undoubtedly influence therapeutic performance, accumulating evidence demonstrates that mechanical forces are critical and underappreciated determinants of nanoparticle fate throughout the drug delivery cascade [10,12].

**Figure 3:**
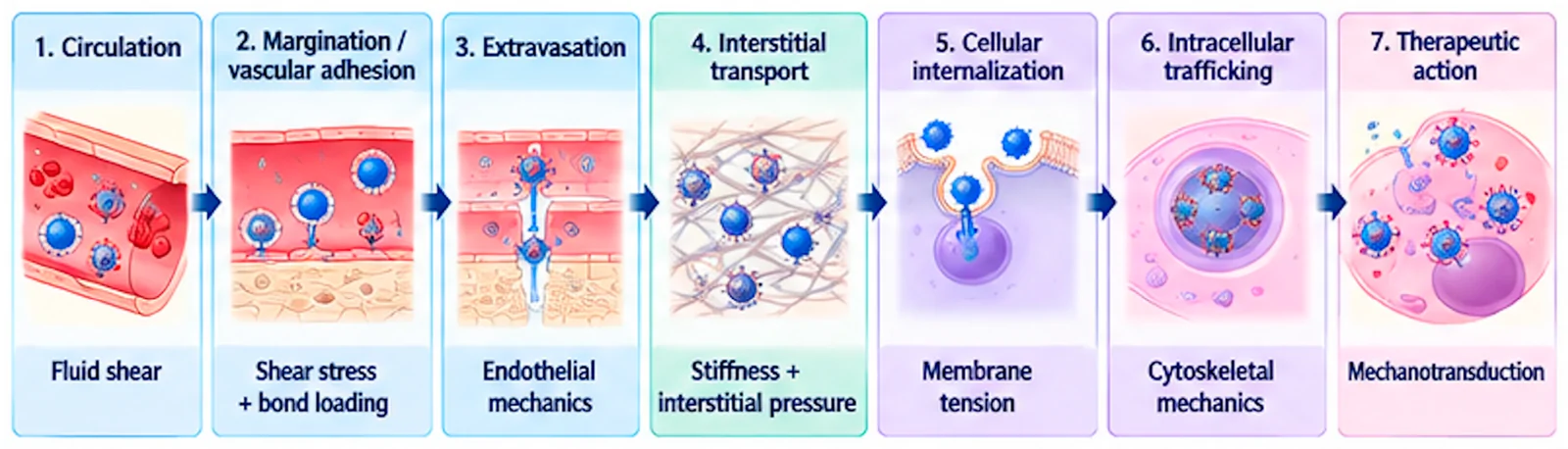
Mechanical regulation across the nanotherapeutic delivery cascade. Following systemic administration, nanotherapeutics encounter sequential mechanical environments that influence circulation, margination and adhesion, extravasation, interstitial penetration, cellular internalization, intracellular trafficking, and therapeutic action. The dominant physical determinants change across the pathway, from fluid shear and hydrodynamic interactions in the vasculature to extracellular-matrix mechanics, interstitial pressure, membrane tension, and cytoskeletal transport at tissue and cellular scales.

From circulation and vascular adhesion to tissue penetration and cellular uptake, nanoparticles encounter dynamically changing mechanical environments that regulate transport efficiency, biodistribution, and therapeutic efficacy. These environments are characterized by complex interactions among fluid forces, vascular mechanics, ECM architecture, cellular contractility, and tissue-specific mechanical properties. Consequently, nanoparticle delivery should be viewed not solely as a biochemical process but as a mechanobiological transport problem operating across multiple spatial and temporal scales. Understanding how mechanical forces regulate nanoparticle behavior is therefore essential for the rational design of next-generation nanotherapeutics. These processes are organized schematically in Figure 3, which illustrates how mechanical cues influence nanotherapeutic fate across the delivery cascade.

### 3.1. Circulatory Transport and Margination

The circulatory system serves as the primary transport network for systemically administered nanotherapeutics. Upon intravenous administration, nanoparticles immediately enter a mechanically dynamic environment dominated by blood flow, hydrodynamic forces, vessel geometry, and interactions with circulating blood cells.

Blood flow generates fluid shear stresses that directly influence nanoparticle motion, orientation, aggregation, and transport behavior [54,55]. The magnitude of these forces varies considerably throughout the vascular tree, ranging from relatively low shear stresses within venous circulation to substantially higher values in arteries and regions of vascular stenosis. Shear forces affect nanoparticle trajectories through hydrodynamic interactions with erythrocytes, leukocytes, platelets, and vessel walls. Under flow conditions, nanoparticles do not simply travel passively with the bloodstream. Instead, their movement reflects complex force balances involving drag, lift, diffusion, and particle-cell interactions [56,57]. These dynamics strongly influence circulation times, vascular targeting efficiency, and therapeutic biodistribution.

One of the most important flow-dependent phenomena affecting vascular delivery is margination, the tendency of particles to migrate toward vessel walls [58,59]. In blood flow, deformable red blood cells preferentially occupy the central region of the vessel lumen, creating a cell-free layer adjacent to the endothelial surface [60,61]. Nanoparticles located within this region experience increased opportunities for endothelial interaction and vascular adhesion. Particle margination is strongly influenced by mechanical factors including flow rate, hematocrit, vessel diameter, and particle geometry. Efficient margination increases the probability of successful vascular targeting, whereas poor margination can significantly reduce therapeutic delivery efficiency.

Particle shape represents another critical determinant of transport behavior. While spherical nanoparticles remain the most widely investigated therapeutic platform, numerous studies have demonstrated that elongated, rod-shaped, discoidal, and filamentous particles exhibit distinct hydrodynamic properties [62,63]. Non-spherical particles often display enhanced margination, prolonged circulation times, altered rotational dynamics, and improved vascular interactions compared with conventional spherical systems. Particle geometry therefore directly influences how mechanical forces are experienced and translated into transport outcomes. Particle size, shape, deformability, blood-cell interactions, and local flow therefore jointly determine near-wall transport and vascular encounter probability, making intravascular delivery a coupled particle–fluid mechanics problem rather than a purely chemical targeting problem.

### 3.2. Vascular Adhesion Under Flow

Successful therapeutic delivery frequently requires nanoparticles to adhere to vascular surfaces before crossing endothelial barriers and entering diseased tissues. The process of vascular adhesion occurs within a highly dynamic mechanical environment in which hydrodynamic forces continuously oppose particle attachment.

Blood flow simultaneously promotes and inhibits vascular adhesion [64,65]. Increased flow enhances particle transport toward vessel walls, increasing encounter frequency with endothelial surfaces. However, elevated shear stresses also generate detachment forces that challenge stable adhesion. The balance between these competing effects determines the probability of successful vascular targeting. As a result, adhesion efficiency depends not only on ligand-receptor affinity but also on local hemodynamic conditions.

Targeted nanotherapeutics commonly employ ligands designed to recognize endothelial receptors overexpressed within diseased tissues, including vascular cell adhesion molecule 1 (VCAM-1), intercellular adhesion molecule 1 (ICAM-1), selectins, integrins, and growth factor receptors [6,7]. However, receptor binding within flowing blood differs substantially from static conditions. Mechanical forces alter bond formation rates, bond lifetimes, receptor accessibility, and clustering behavior. Consequently, the performance of targeting ligands must be evaluated within physiologically relevant mechanical environments.

An especially intriguing example of force-regulated adhesion involves catch bonds. Unlike conventional slip bonds that weaken under increasing force, catch bonds exhibit enhanced stability when subjected to moderate tensile loading [66,67]. Several biological adhesion systems exploit catch-bond behavior to maintain stable attachment under flow conditions. Leukocyte rolling, platelet adhesion, and selectin-mediated interactions all involve force-dependent bond stabilization mechanisms [67]. Understanding these principles may enable the development of next-generation vascular targeting strategies that exploit rather than resist mechanical forces.

### 3.3. Extravasation and Interstitial Transport

Following vascular adhesion, nanoparticles must often traverse endothelial barriers and penetrate complex tissue microenvironments before reaching target cells. This stage frequently represents one of the most significant limitations to therapeutic delivery.

Solid tumors present highly abnormal mechanical environments characterized by elevated matrix stiffness, increased solid stress, vascular compression, and abnormal interstitial fluid pressure [15,17]. These mechanical abnormalities significantly influence therapeutic penetration. Increased ECM density restricts diffusion, while elevated tissue stiffness impedes nanoparticle transport through the interstitial space. Simultaneously, solid stresses can collapse blood vessels, reduce perfusion and limiting therapeutic access [15,68]. Consequently, tumor mechanics plays a major role in determining treatment efficacy.

Nanoparticle extravasation is governed by endothelial permeability and vascular barrier integrity. Mechanical forces influence endothelial junctions, cytoskeletal organization, and transvascular transport pathways [34,43]. In tumors and inflamed tissues, altered mechanical environments frequently disrupt endothelial barriers, increasing vascular permeability and facilitating nanoparticle escape into surrounding tissues. However, excessive permeability may also contribute to heterogeneous drug distribution and uneven therapeutic responses.

Once nanoparticles enter tissues, further transport depends upon interstitial mechanics. Interstitial movement occurs through a combination of diffusion and convection and is strongly influenced by ECM density, collagen architecture, tissue stiffness, interstitial fluid pressure, and hydraulic conductivity [13]. Dense and highly crosslinked matrices frequently reduce nanoparticle penetration, producing steep concentration gradients that limit therapeutic efficacy [10,14]. The mechanical architecture of tissues therefore represents a critical determinant of local drug distribution.

### 3.4. Mechanics of Cellular Internalization

Ultimately, successful therapeutic delivery requires nanoparticle uptake by target cells. Cellular internalization is increasingly recognized as a mechanically regulated process rather than a purely biochemical event.

Endocytosis involves substantial membrane deformation and therefore requires mechanical work [16,20]. The energetic cost of membrane bending depends upon membrane tension, cortical stiffness, cytoskeletal organization, and local mechanical conditions. Increased membrane tension can suppress endocytic activity, whereas reductions in membrane tension may facilitate nanoparticle internalization [69]. Consequently, the efficiency of nanoparticle uptake is strongly influenced by the mechanical state of the cell.

The cytoskeleton plays a central role in force generation and intracellular transport. Actin filaments, microtubules, and molecular motor proteins regulate membrane remodeling, vesicle formation, intracellular trafficking, and endosomal transport [18,19,70]. Mechanical perturbations that alter cytoskeletal organization can profoundly influence nanoparticle uptake pathways and intracellular fate. Furthermore, matrix stiffness and cellular contractility regulate endocytic machinery through mechanotransduction pathways involving integrins, focal adhesions, Rho GTPases, and YAP/TAZ signaling [38,40]. As a result, cells residing in mechanically distinct environments may exhibit dramatically different therapeutic uptake efficiencies even when exposed to identical nanoparticle formulations.

### 3.5. Engineered Nanostructure–Cell Interfacial Mechanics

Mechanically mediated intracellular delivery is not limited to the endocytosis of freely suspended nanoparticles. A distinct nano–bio interfacial mechanism occurs when engineered surface nanostructures interact directly with the plasma membrane. High-aspect-ratio nanoneedles can concentrate mechanical loading over nanoscale contact regions, producing membrane deformation or transient nanoperforation that enables molecular cargo to enter the cytosol without relying exclusively on conventional receptor-mediated uptake.

This contact-based delivery mechanism has been demonstrated using diamond nanoneedle arrays applied to cells under controlled centrifugation-induced loading. The interaction forces were on the order of a few nanonewtons, allowing transient membrane deformation and delivery of diverse molecular cargo, including DNA and nanoparticles. In primary neurons, the nanoneedle approach produced approximately 45% plasmid-DNA transfection compared with approximately 1–5% for conventional lipofection, demonstrating that nanoscale contact mechanics can directly alter intracellular delivery efficiency [71].

More recently, nanoneedle mechanics have been integrated with microfluidic transport. A vibration-assisted nanoneedle/microfluidic platform was developed to mechanically puncture rapidly moving suspension cells and enable continuous intracellular delivery. The system achieved delivery efficiencies of up to 98% in difficult-to-transfect immune cells while maintaining approximately 80% cell viability and operating at throughput on the order of mL∙min^−1^ [72].

These studies extend mechanics-guided nanotherapeutics beyond materials that passively encounter a mechanical environment. In nanostructure–cell interfaces, the engineered nanoscale architecture itself generates and localizes mechanical interactions at the membrane. Relevant design parameters therefore include nanostructure diameter, height, spacing, contact force, membrane tension, cortical stiffness, loading rate, and interaction time. Quantitative relationships among these variables will be required to maximize intracellular access while maintaining membrane recovery and cell viability. Such interfaces provide a direct connection among nanofabrication, interfacial mechanics, microfluidic engineering, and therapeutic delivery.

## 4. Mechanically Responsive Nanotherapeutics

Mechanically responsive therapeutic systems use a physical loading condition or mechanically dependent material state to alter carrier structure, transport, cargo exposure, or therapeutic release. This strategy differs from conventional stimuli-responsive nanomedicine, in which activation is typically governed by pH, enzymes, redox chemistry, temperature, or other biochemical signals [8,17,35,73].

The experimental maturity of mechanics-responsive systems is highly uneven. Shear-activated vascular delivery provides the clearest direct demonstration of a pathological mechanical cue being converted into a nanotherapeutic activation criterion. By contrast, stiffness-, strain-, and pressure-responsive concepts remain substantially less developed, and much of the supporting literature involves mechanosensitive biomaterials, hydrogels, or theoretical design opportunities rather than validated disease-triggered nanotherapeutics. The discussion below therefore distinguishes experimentally demonstrated systems from emerging concepts and avoids treating all mechanical-response categories as equally established.

**Figure 4:**
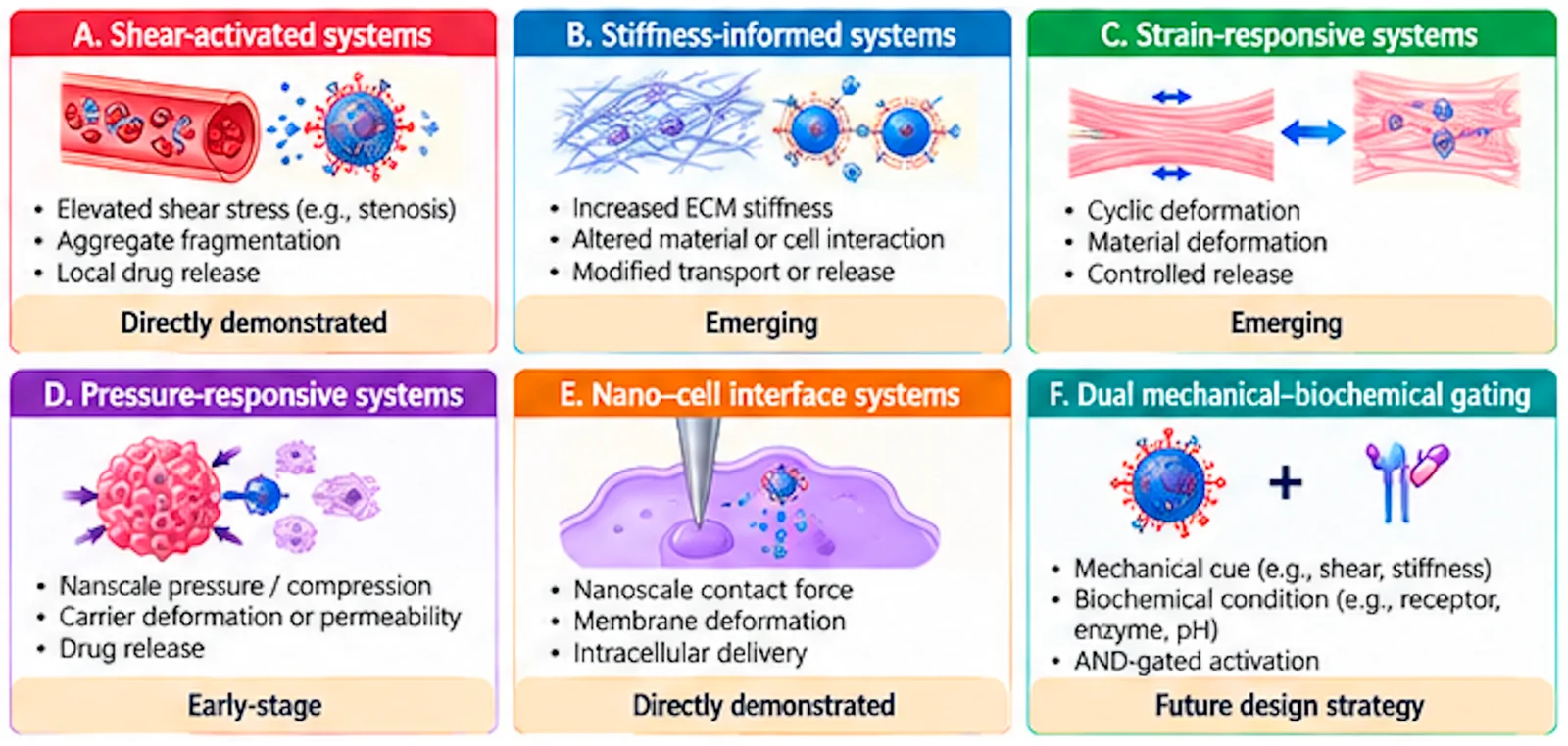
Mechanically responsive and interface-mediated therapeutic strategies. Mechanical cues can influence therapeutic behavior through shear-induced carrier fragmentation, stiffness-dependent material interactions, strain-induced deformation, pressure- or compression-dependent material response, or direct nanoscale contact with cellular membranes. The maturity of these approaches differs substantially; shear-activated vascular systems and nanoneedle-mediated intracellular delivery have direct experimental demonstrations, whereas several stiffness-, strain-, pressure-, and dual-gated strategies remain at earlier stages of development.

For therapeutic design, the important question is not simply whether a disease exhibits abnormal mechanics, but whether that abnormality can be converted into a reproducible operating window that separates the target condition from normal physiological exposure. Figure 4 and Table 1 summarize the principal mechanical-response strategies, their activation mechanisms, representative evidence, and current stage of development.

### 4.1. Shear-Activated Vascular Nanotherapeutics: A Quantitative Design Example

Shear-activated vascular delivery provides one of the most direct demonstrations of mechanics being used as a therapeutic design variable rather than merely described as a biological phenomenon. Korin et al. developed micron-scale aggregates composed of smaller nanoparticles that remain intact during ordinary circulation but fragment when exposed to the elevated shear generated by severe vascular narrowing [74]. The design was inspired by the ability of platelets to respond preferentially to high-shear regions of obstructed vessels. Rather than relying only on receptor expression, the carrier architecture converts a pathological hydrodynamic environment into a physical disassembly mechanism.

The key engineering principle is the existence of a differential shear regime between unobstructed and severely narrowed vessels. As blood accelerates through a stenosis, local shear can increase markedly relative to normal vascular conditions. The shear-activated aggregates were engineered so that hydrodynamic forces in these high-shear regions exceeded the cohesive interactions maintaining aggregate structure, thereby releasing nanoscale therapeutic components locally at the obstruction [74]. This represents an explicit force–response design relationship: carrier stability is maintained below the activation regime, whereas suprathreshold hydrodynamic loading produces fragmentation and localized therapeutic deployment.

This example provides a useful quantitative design template for mechanics-guided therapeutics. A mechanical system should ideally be characterized by at least four measurable quantities: the physiological mechanical exposure distribution, the pathological exposure distribution, the material activation threshold, and the width of the transition between inactive and activated states. These quantities determine whether a useful therapeutic window exists. Importantly, the same principle also reveals a major translational limitation: activation cannot be assumed to be disease-specific unless the pathological mechanical exposure is sufficiently separated from transient physiological extremes.

At present, thrombotic vascular obstruction remains the most established application of this strategy. Extension to other cardiovascular disorders should therefore be described as a design opportunity rather than an already validated therapeutic class. Conditions such as restenosis, vascular inflammation, or device-associated flow disturbances may provide suitable mechanical environments, but each application will require direct measurement of local hemodynamics and validation of a corresponding activation window.

### 4.2. Stiffness-Informed and Stiffness-Responsive Strategies

Tissue stiffness is a prominent physical feature of fibrosis and many solid tumors, but direct stiffness-triggered nanotherapeutic systems remain less established than shear-activated platforms. Increased rigidity can nevertheless influence therapeutic performance indirectly by altering extracellular-matrix architecture, cell contractility, endocytosis, and mechanotransduction [17,29,30,31,32,38,40]. Stiffness should therefore be considered first as a tissue property that modifies transport and cellular response, and only secondarily as a potential activation variable.

Mechanically dynamic hydrogels and other biomaterials provide proof that material behavior can be coupled to changes in local mechanics. Permeability, degradation, molecular exposure, ligand presentation, or release kinetics can in principle be engineered to depend on deformation, crosslinking state, or force transmission [36,74,75]. However, these systems should not be interpreted as evidence that clinically established stiffness-triggered nanotherapeutics already exist. A key future requirement is to demonstrate that the mechanical property measured in a diseased tissue produces a reproducible material response that does not occur across the corresponding healthy-tissue stiffness distribution.

Accordingly, future stiffness-responsive designs may involve mechanically activated molecular switches, ECM-interacting particles, force-sensitive supramolecular assemblies, or materials that change transport or release behavior according to matrix mechanics. Such systems will require quantitative calibration of material response against clinically measured stiffness distributions before stiffness can function as a reliable therapeutic trigger.

### 4.3. Strain-Responsive Strategies

Strain differs fundamentally from stiffness because it represents deformation rather than an intrinsic material property. Cyclic deformation occurs in the heart, lungs, vasculature, musculoskeletal system, and other dynamically loaded tissues. This makes strain conceptually attractive as a temporal input for therapeutic materials, particularly where disease changes the amplitude, spatial distribution, or frequency of tissue deformation [35,76].

Existing evidence is strongest in mechanically active biomaterials and regenerative systems rather than in mature nanotherapeutic platforms. Stretch-sensitive polymers, deformable hydrogels, mechanically adaptive scaffolds, and force-sensitive molecular assemblies illustrate ways in which cyclic loading could alter permeability, molecular conformation, or release. Potential applications include post-infarction cardiac remodeling, musculoskeletal repair, vascular grafts, and mechanically active tissue interfaces.

For translation into strain-triggered nanotherapeutics, however, the material response must be calibrated against both normal physiological cycling and pathological deformation. Activation based simply on the presence of strain would not be selective because healthy organs such as the heart and lungs undergo continuous deformation. Useful systems will therefore require response thresholds, frequency dependence, fatigue resistance, or combined biochemical gating that distinguishes disease-associated loading from normal physiological motion.

### 4.4. Pressure- and Compression-Responsive Strategies

Hydrostatic pressure, interstitial fluid pressure, and solid compressive stress can alter both therapeutic transport and material deformation. Elevated interstitial fluid pressure in solid tumors is particularly important because it suppresses convective transport and contributes to heterogeneous nanoparticle penetration [13,14,15,17]. Pressure therefore has an established role as a transport barrier, whereas its direct use as a nanotherapeutic activation trigger remains comparatively immature.

Prospective pressure-responsive systems could use deformation-sensitive carriers, mechanosensitive polymers, pressure-dependent permeability, or mechanically induced structural transitions. However, direct evidence linking clinically measured pathological pressure ranges to reproducible therapeutic activation remains limited. These systems should therefore be considered an emerging design direction rather than an established therapeutic category.

Translation will require demonstration that the relevant pressure or compressive-stress range can be measured in vivo, reproduced experimentally, and separated sufficiently from normal physiological fluctuations to generate a safe activation window. Combining pressure sensitivity with biochemical targeting may be particularly important when mechanical distributions overlap between healthy and diseased tissues.

## 5. Self-Assembled Nanotherapeutics and Supramolecular Systems

Among the numerous nanotherapeutic platforms currently under development, self-assembled supramolecular systems occupy a unique position at the interface of materials science, nanotechnology, and molecular medicine [77,78]. Unlike conventional nanoparticles that are often fabricated through top-down manufacturing approaches, supramolecular nanotherapeutics emerge through spontaneous molecular organization driven by noncovalent interactions, including hydrogen bonding, hydrophobic interactions, electrostatic forces, van der Waals interactions, and π–π stacking [79,80]. These interactions enable the formation of highly ordered nanoscale architectures capable of dynamic adaptation, reversible assembly, and environmentally responsive behavior. Among existing nanotherapeutic platforms, supramolecular systems are especially well suited for mechanics-guided design because their structure and function arise from reversible intermolecular interactions that are inherently sensitive to environmental perturbation.

The inherent reversibility of supramolecular systems distinguishes them from many traditional drug delivery platforms. Because assembly is governed by weak intermolecular interactions rather than permanent covalent bonds, these materials exist in a delicate thermodynamic balance between assembled and disassembled states [81]. Consequently, supramolecular nanostructures are uniquely positioned to respond to changes in their local physical and biochemical environment.

This dynamic behavior has generated significant interest in peptide-based nanotherapeutics, self-assembled drug depots, injectable supramolecular materials, and biomimetic delivery systems. Importantly, many of these systems possess properties that make them particularly attractive for integration with mechanobiological concepts. Unlike rigid nanoparticles, supramolecular assemblies can undergo force-dependent structural reorganization, mechanically regulated disassembly, and adaptive changes in molecular packing. These characteristics suggest that self-assembled nanotherapeutics may provide a natural platform for future mechanically responsive drug delivery systems.

### 5.1. Peptide Self-Assembly as a Therapeutic Platform

Peptides represent one of the most versatile building blocks for supramolecular nanomedicine [82,83]. Their molecular programmability, biocompatibility, biodegradability, and ability to access diverse conformational states enable the formation of a wide variety of nanoscale structures, including nanofibers, fibrils, nanotubes, vesicles, micelles, hydrogels, and hierarchical supramolecular networks [84]. Through careful sequence design, peptide assemblies can be engineered to control mechanical properties, molecular stability, degradation kinetics, cargo retention, and biological activity [78,85].

A particularly attractive feature of peptide self-assembly is the ability to create high-density drug depots composed entirely of bioactive molecules. In such systems, therapeutic peptides serve simultaneously as the drug and the structural material, eliminating the need for additional carriers and maximizing therapeutic loading [86,87]. These self-delivering systems have emerged as promising strategies for the sustained administration of peptide therapeutics in metabolic disease, regenerative medicine, and chronic disorders [88].

Beyond therapeutic delivery, peptide self-assembly also provides opportunities to investigate fundamental relationships between molecular structure, supramolecular organization, and biological function. Small modifications in amino acid sequence can dramatically alter assembly pathways, fibril morphology, mechanical properties, and release behavior, highlighting the remarkable tunability of these systems.

### 5.2. Supramolecular Drug Depots

Long-acting therapeutics remain one of the major challenges in modern medicine [89]. Conventional formulations often require repeated administration because of rapid drug clearance, enzymatic degradation, or poor tissue retention. Self-assembled supramolecular depots provide an alternative strategy in which therapeutic molecules are temporarily stored within highly ordered nanoscale assemblies that gradually release active species over time.

The release kinetics of such systems are governed by a dynamic equilibrium between assembled and free molecular states [79,90]. Drug release therefore depends on multiple interconnected factors, including assembly thermodynamics, molecular packing, fibril stability, monomer dissociation kinetics, and local environmental conditions. This mechanism differs fundamentally from conventional diffusion-controlled delivery systems. Rather than acting as passive containers, supramolecular depots function as dynamic molecular reservoirs whose behavior is intrinsically linked to the stability of the assembled state [78].

Several peptide therapeutics currently under clinical investigation exploit self-assembly to achieve prolonged biological activity [91,92,93,94]. These systems illustrate how supramolecular organization can be leveraged to overcome limitations associated with rapid drug degradation and clearance. Importantly, because supramolecular assemblies exist near thermodynamic equilibrium, they may be particularly sensitive to mechanical perturbations capable of altering assembly stability and release behavior.

### 5.3. Dynamic Fibrillar Systems

Among self-assembled nanostructures, fibrillar systems are especially interesting from a mechanobiological perspective. Fibrils represent highly anisotropic architectures composed of molecular subunits arranged through cooperative self-assembly processes. Depending on molecular design and environmental conditions, fibrils may exhibit diverse morphologies, including twisted ribbons, helical filaments, bundled fibers, and hierarchical networks. Traditionally, fibrillar drug depots have been viewed as relatively static storage structures. However, increasing evidence suggests that fibrillar assemblies should instead be regarded as dynamic systems undergoing continuous molecular exchange with their surroundings [90,95].

At equilibrium, monomer association and dissociation occur simultaneously at fibril interfaces [96,97]. Consequently, fibrils possess the capacity to adapt to environmental changes through alterations in molecular packing, assembly stability, and growth behavior. This dynamic nature provides opportunities for the development of therapeutic systems capable of responding to external stimuli, including mechanical forces. From a nanomedicine perspective, fibrils may therefore represent more than simple drug reservoirs; they may function as adaptive supramolecular materials capable of integrating environmental information into therapeutic behavior.

### 5.4. Mechanically Influenced Assembly

Although biochemical factors regulating self-assembly have been extensively studied, the role of mechanical forces remains comparatively underexplored. In biological environments, supramolecular assemblies are continuously exposed to a wide range of mechanical stimuli, including fluid shear stress, cyclic strain, tissue deformation, matrix confinement, interstitial flow, and cellular traction forces [98]. These mechanical cues can influence assembly pathways by modifying molecular transport, collision frequencies, conformational states, and intermolecular interactions.

Fluid shear, for example, has long been recognized as a regulator of protein aggregation and fibrillar growth [99,100]. Hydrodynamic forces can alter nucleation kinetics, accelerate fibril fragmentation, generate new growth interfaces, and influence supramolecular morphology [90,101]. Similarly, mechanical deformation may affect molecular packing within assembled structures by altering intermolecular spacing and local free-energy landscapes. Such observations suggest that supramolecular assembly should not be viewed solely as a chemically controlled process. Rather, assembly pathways may emerge from a complex interplay between thermodynamic driving forces and mechanical influences. Understanding these interactions represents an important frontier for the development of mechanically responsive biomaterials. Importantly, these effects suggest that mechanics may influence supramolecular assembly not only by changing molecular transport, but also by reshaping the free-energy landscape that governs molecular organization.

### 5.5. Force-Mediated Dissociation and Therapeutic Release

While much attention has focused on force-induced assembly processes, mechanical forces may also regulate supramolecular disassembly. Because supramolecular architectures are stabilized through noncovalent interactions, externally applied forces have the potential to alter assembly stability by modifying intermolecular energy barriers. Mechanical perturbations may therefore influence monomer release, fibril fragmentation, structural transitions, cargo liberation, and depot erosion [90,102]. Force-mediated dissociation introduces a particularly intriguing possibility for drug delivery.

Rather than relying exclusively on biochemical triggers such as pH or enzymatic degradation, therapeutic release could be regulated through mechanical interactions occurring within diseased tissues. For example, pathological environments characterized by abnormal flow, elevated strain, altered stiffness, or increased pressure may generate mechanical conditions capable of accelerating disassembly and enhancing local drug release. Although experimental demonstrations remain limited, this concept provides a compelling framework for future research at the intersection of supramolecular chemistry, mechanobiology, and nanomedicine. Establishing quantitative relationships among applied force, supramolecular stability, dissociation kinetics, and therapeutic release remains a key requirement for transforming this concept into a predictive design strategy.

## 6. Mechanobiology and Therapeutic Targeting

Mechanotransduction pathways provide a direct molecular bridge between disease-associated mechanics and therapeutic intervention. While traditional biology largely viewed cellular behavior through the lens of biochemical signaling networks, it is now recognized that cells continuously integrate both molecular and mechanical information to regulate physiological function [22,24,35]. Mechanical forces generated by fluid flow, matrix stiffness, tensile strain, compression, and tissue deformation are detected through specialized mechanosensory systems and converted into biochemical responses through mechanotransduction pathways [22].

Importantly, these pathways do not merely respond to mechanical cues; they actively regulate cellular proliferation, migration, differentiation, metabolism, inflammation, fibrosis, and survival. Dysregulation of mechanotransduction has been implicated in numerous pathological conditions including cancer, cardiovascular disease, fibrosis, pulmonary hypertension, neurodegeneration, and metabolic disorders [21,24]. Consequently, mechanotransduction machinery has emerged as a promising but still relatively underexplored class of therapeutic targets.

For nanomedicine, this presents an intriguing opportunity. Rather than focusing exclusively on molecular biomarkers, therapeutic systems may be designed to directly target mechanosensory pathways that govern disease progression. Such an approach would bridge mechanobiology and drug delivery, enabling interventions directed toward the physical mechanisms that underlie pathological remodeling.

Central to this concept is the mechanotransduction machinery that converts physical forces into biochemical responses. Mechanosensors, force-sensitive signaling networks, and downstream transcriptional regulators collectively integrate mechanical information and govern cellular adaptation to changing physical environments.

### 6.1. PIEZO1: Mechanosensing and Therapeutic Relevance

Among recently discovered mechanosensors, PIEZO1 has emerged as one of the most important mediators of cellular force transduction. PIEZO1 is a mechanically activated ion channel that converts membrane tension into intracellular calcium signaling. Activation occurs in response to diverse mechanical stimuli including fluid shear stress, membrane stretch, hydrostatic pressure, mechanical deformation, and cell crowding [26,27,28]. Following mechanical activation, PIEZO1 opens a nonselective cation channel that permits rapid calcium influx and initiates downstream signaling cascades.

PIEZO1 plays critical roles in vascular biology and cardiovascular homeostasis. Endothelial PIEZO1 contributes to flow sensing, nitric oxide production, vascular remodeling, angiogenesis, and blood pressure regulation [103,104,105]. Loss of PIEZO1 function disrupts endothelial mechanosensing and impairs normal vascular adaptation to hemodynamic forces.

Aberrant PIEZO1 signaling has been implicated in atherosclerosis, hypertension, pulmonary vascular disease, cancer progression, and fibrotic remodeling [106,107]. Because PIEZO1 functions at the interface between mechanical stimuli and cellular signaling, it represents an attractive therapeutic target for diseases characterized by abnormal physical environments.

Future nanotherapeutics may exploit PIEZO1 through several strategies, including targeted delivery of PIEZO1 modulators, mechanically activated drug release systems, gene regulation approaches, and tissue-specific mechanosensory interventions. Such systems could directly modulate force-sensing pathways rather than targeting downstream consequences of mechanotransduction.

### 6.2. PIEZO2: Context-Dependent Therapeutic Potential

PIEZO2 represents a second major member of the PIEZO family and serves as a principal mechanosensor in numerous physiological systems [26]. While PIEZO1 is broadly involved in vascular and systemic mechanotransduction, PIEZO2 is particularly important for touch sensation, proprioception, mechanical pain, stretch sensing, and sensory neuron function [26,108,109]. Recent studies increasingly suggest that PIEZO2 may also contribute to mechanoregulation in non-neuronal tissues.

Emerging evidence suggests possible roles for PIEZO2 beyond classical sensory mechanotransduction, including context-dependent contributions to cardiovascular development, tissue remodeling, and fibrotic signaling; however, these functions remain considerably less established than those of PIEZO1 [27,28]. Because PIEZO2 mediates specialized mechanical responses, its therapeutic relevance may be greatest in neurological, pain-related, sensory, and selected mechanically regulated disease contexts.

### 6.3. Integrin-Focal Adhesion Signaling

Whereas PIEZO channels directly sense membrane tension, integrins function as primary mediators of cell-ECM interactions. Integrins are transmembrane receptors that physically connect ECM proteins to intracellular cytoskeletal structures [38]. This architecture allows forces generated within tissues to be transmitted directly into the cell. Mechanical signals received through integrins regulate cell adhesion, migration, survival, differentiation, and gene expression.

Integrin-mediated forces are transmitted through focal adhesions, highly dynamic multiprotein assemblies that serve as mechanical communication hubs [39]. Key focal adhesion components include talin, vinculin, paxillin, focal adhesion kinase (FAK), and Src family kinases [39,110]. Mechanical loading induces conformational changes within these proteins, exposing cryptic binding sites and activating downstream signaling pathways.

Abnormal integrin signaling contributes to tumor invasion, metastasis, fibrosis, vascular remodeling, and inflammatory disease [38,111]. Because integrins lie upstream of numerous mechanobiological pathways, they offer attractive opportunities for therapeutic intervention.

Nanomedicine approaches targeting integrins have already demonstrated promise in tumor targeting, anti-angiogenic therapy, fibrosis treatment, and regenerative medicine. Future strategies may move beyond simple receptor targeting and instead directly modulate integrin-mediated mechanotransduction.

### 6.4. YAP/TAZ Mechanotrasduction

One of the most influential discoveries in modern mechanobiology has been the identification of YAP and TAZ as central transcriptional regulators of mechanical signaling [40]. YAP and TAZ (transcriptional coactivator with PDZ-binding motif) act as mechanosensitive transcriptional co-regulators that integrate information from matrix stiffness, cell geometry, cytoskeletal tension, cell density, and mechanical loading [40,41]. Unlike membrane-associated mechanosensors, YAP/TAZ function within the nucleus, where they directly regulate gene expression.

Soft mechanical environments generally suppress YAP/TAZ activity, whereas increased stiffness and cytoskeletal tension promote nuclear localization and transcriptional activation [40,112]. Activated YAP/TAZ regulate genes associated with proliferation, survival, fibrosis, stemness, and tumor progression.

Aberrant YAP/TAZ signaling has been implicated in cancer, organ fibrosis, cardiac remodeling, pulmonary hypertension, and tissue regeneration [41,113,114]. Given their central role in converting mechanical information into transcriptional responses, YAP/TAZ represent highly attractive mechanotherapeutic targets.

Nanotherapeutic systems may facilitate targeted delivery of YAP/TAZ inhibitors, RNA interference approaches, gene editing technologies, and combination mechanobiology therapies. Such approaches could directly interrupt mechanical signaling pathways that drive disease progression.

### 6.5. Cytoskeletal Pathways and Mechanical Signal Integration

The cytoskeleton represents the physical infrastructure through which cells generate, transmit, and respond to forces. Actin filaments, microtubules, and intermediate filaments collectively form a dynamic network that governs cell shape, force generation, migration, intracellular transport, and mechanotransduction [18,115]. Importantly, the cytoskeleton functions not merely as a structural scaffold but as an active participant in mechanical signaling. From a design perspective, the cytoskeleton represents not only a biological target but also an intracellular transport infrastructure that nanotherapeutics must successfully navigate.

Mechanical forces sensed at the cell surface are transmitted through cytoskeletal networks to intracellular organelles and the nucleus [115]. This process enables rapid communication between extracellular mechanical stimuli and intracellular signaling machinery [115,116].

Abnormal cytoskeletal organization contributes to cancer metastasis, fibrosis, vascular disease, cardiomyopathy, and neurodegeneration [18,24]. Changes in cellular contractility frequently amplify disease-associated mechanical abnormalities.

Nanotherapeutic design should account for the fact that cytoskeletal mechanics regulate both cellular entry and intracellular fate. Actin remodeling influences membrane wrapping and endocytic vesicle formation, whereas microtubules and motor proteins guide endosomal trafficking, perinuclear transport, and intracellular distribution. Thus, cytoskeletal state may determine whether internalized nanotherapeutics reach productive intracellular compartments or remain trapped in non-therapeutic trafficking pathways.

### 6.6. Mechanotransduction as a Therapeutic Target

Mechanotransduction machinery represents an emerging therapeutic target class because it lies upstream of many pathological biochemical responses. Rather than intervening only after downstream inflammatory, fibrotic, proliferative, or remodeling pathways have been activated, mechanotherapeutic strategies could modulate the force-sensing systems that initiate these programs [24,27,38,41].

This upstream positioning creates opportunities for intervention at multiple mechanobiological levels, including mechanosensitive ion channels, integrin–focal adhesion complexes, FAK/Src signaling, Ras homolog family member A (RhoA)/Rho-associated coiled-coil-containing protein kinase (ROCK)-mediated contractility, YAP/TAZ transcriptional regulation, and cytoskeletal remodeling. Among these candidates, PIEZO1, integrin-associated signaling, FAK/Src, RhoA/ROCK, and YAP/TAZ currently have the strongest mechanistic foundation for therapeutic exploration, whereas PIEZO2-targeted strategies require more context-specific validation.

## 7. Prospective Opportunities for Mechanics-Informed Precision Nanomedicine

The concepts discussed in this section remain prospective and should not be interpreted as established mechanics-guided nanotherapeutic capabilities. Their rationale arises from the convergence of patient-specific biomechanical measurement, computational modeling, nanomedicine, medical imaging, and machine learning. These technologies currently exist primarily as parallel fields rather than as fully integrated mechanics-guided therapeutic platforms.

Their potential relevance follows from a practical problem: mechanical properties vary across patients, lesions, anatomical locations, disease stages, and treatment histories. Consequently, a mechanically responsive formulation calibrated against a single nominal stiffness, shear stress, pressure, or strain value may not perform consistently across a heterogeneous patient population. Future computational approaches could help convert these distributions into patient-specific design constraints, but this remains a research objective rather than a current clinical capability.

### 7.1. Mechanics as an Information Layer in Precision Medicine

Precision medicine has traditionally relied on molecular information, including genomic, proteomic, metabolic, and imaging-derived biomarkers. Mechanics adds a complementary physical information layer. Tissue stiffness, vascular shear profiles, strain distributions, pressure gradients, and viscoelastic properties can encode disease stage, tissue remodeling, transport barriers, and therapeutic vulnerability. Integrating these mechanical signatures with molecular biomarkers may enable more complete patient stratification and more accurate prediction of therapeutic response. For mechanics-guided nanomedicine, this shift is especially important because therapeutic performance depends not only on molecular targeting but also on the physical environments encountered during circulation, tissue penetration, cellular uptake, and intracellular trafficking. Mechanical measurements may therefore provide complementary variables for therapeutic stratification when they add predictive information beyond molecular and anatomical biomarkers.

### 7.2. Prospective Role of Machine Learning

Direct demonstrations in which machine learning is used specifically to optimize a mechanically responsive nanotherapeutic remain limited. Machine learning should therefore be viewed here as a prospective enabling tool rather than an established component of mechanics-guided nanomedicine.

A plausible role is the analysis of multidimensional mechanical datasets in which stiffness, shear stress, pressure, strain, anatomy, carrier properties, and therapeutic outcomes vary simultaneously. Models trained on sufficiently large and experimentally validated datasets could potentially identify combinations of mechanical variables associated with carrier transport, activation, or response. Such models could also assist in defining activation windows when healthy and diseased mechanical distributions overlap.

The principal limitation is data quality rather than algorithm availability. Mechanics-guided machine learning will require standardized measurements, explicit mechanical units and boundary conditions, experimentally measured therapeutic outcomes, and external validation across independent biological systems. Until such datasets exist, AI-based optimization of mechanics-responsive nanotherapeutics should remain framed as a future research direction.

### 7.3. Digital Twins as a Future Multiscale Framework

Patient-specific digital twins that couple tissue mechanics directly to mechanics-responsive nanotherapeutic behavior are not yet an established clinical technology. The concept is nevertheless relevant because several constituent capabilities already exist independently, including patient-specific computational fluid dynamics, finite-element models of tissue deformation, image-based biomechanical reconstruction, pharmacokinetic modeling, and computational drug-transport analysis.

A future mechanics-informed digital twin would require these components to be integrated rather than merely collocated. For example, patient-specific vascular geometry could provide local wall-shear and flow fields, tissue imaging could constrain stiffness or deformation distributions, and a validated carrier model could predict margination, extravasation, activation, or tissue penetration under those conditions. Such a framework would then require prospective comparison between model predictions and measured therapeutic outcomes.

Accordingly, digital twins are presented in this Perspective as a testable future framework. Their value would be demonstrated only if incorporating mechanical information significantly improves prediction of therapeutic localization, response, or toxicity compared with models based on anatomy, pharmacokinetics, and molecular biomarkers alone.

### 7.4. Patient-Specific Mechanics and Therapeutic Stratification

A fundamental limitation of many contemporary therapeutic strategies is the implicit assumption that patients with the same clinical diagnosis exhibit similar biological and biophysical characteristics. Increasing evidence suggests that this assumption is often incorrect. In addition to molecular heterogeneity, patients display substantial variability in the mechanical properties of tissues, organs, and vascular systems. These differences arise from age, sex, genetic background, disease stage, inflammatory status, ECM remodeling, lifestyle factors, and environmental exposures. Consequently, individuals with apparently similar clinical presentations may possess markedly distinct mechanical microenvironments that influence disease progression, therapeutic transport, and treatment response.

Mechanical heterogeneity is now recognized across numerous disease states. In cancer, tumor stiffness can vary by more than an order of magnitude among patients and even within different regions of the same lesion, reflecting differences in ECM composition, stromal activation, vascular architecture, and cellular contractility. Fibrotic diseases similarly exhibit patient-specific patterns of matrix remodeling and tissue stiffening that influence both disease progression and therapeutic accessibility. In cardiovascular disorders, variations in vessel geometry, arterial compliance, wall shear stress, and blood-flow patterns strongly affect vascular remodeling and clinical outcomes. Likewise, pulmonary hypertension encompasses a spectrum of mechanically distinct vascular phenotypes characterized by heterogeneous patterns of pressure overload, vessel stiffening, and pulmonary vascular remodeling. These observations suggest that mechanical signatures may provide clinically meaningful information that complements traditional molecular and histopathological biomarkers.

For mechanics-guided nanomedicine, this heterogeneity means that activation thresholds cannot be assumed to be universal. A stiffness-responsive system optimized for one tumor type, disease stage, or anatomical location may not respond appropriately in another mechanically distinct microenvironment.

Recent advances in biomechanical characterization are creating new opportunities to quantify patient-specific mechanical phenotypes in vivo. Techniques such as magnetic resonance elastography, ultrasound elastography, optical coherence elastography, computational fluid dynamics, and emerging biomechanical sensing technologies increasingly enable the noninvasive assessment of tissue stiffness, hemodynamic forces, pressure distributions, and deformation patterns across multiple biological scales. As these approaches mature, mechanical measurements may become routine components of clinical evaluation, providing quantitative descriptions of the physical microenvironments in which therapeutics operate.

The translational value of mechanical phenotyping will depend on whether these measurements improve therapeutic decisions. Potential uses include selection of delivery route, carrier architecture, dosing strategy, or activation threshold according to patient-specific stiffness, flow, pressure, or deformation. However, mechanical measurements should not be assumed to improve precision medicine automatically. Their value must be established prospectively by demonstrating additional predictive or therapeutic benefit beyond existing molecular, anatomical, and clinical variables.

### 7.5. Long-Term Concept: Mechanically Adaptive Therapeutics

A longer-term objective is the development of therapeutics whose behavior changes dynamically with the mechanical state of diseased tissue. Such systems could, in principle, alter release, carrier stability, or biological activity as stiffness, shear, pressure, or strain evolves during disease or treatment.

A closed-loop version of this concept would require therapeutic activation to decrease as the pathological mechanical stimulus is normalized. This remains hypothetical. Demonstration would require a defined mechanical input, a reproducible force–response relationship, evidence that therapeutic action modifies the relevant mechanical state, and reversible or self-limiting reduction in activation as that state returns toward a physiological range.

## 8. Mechanics-Specific Challenges for Clinical Translation

Clinical translation of mechanically responsive therapeutics introduces requirements that are not captured fully by conventional nanomedicine development. In addition to composition, pharmacokinetics, drug loading, and toxicity, these systems require characterization of a force–response relationship. Relevant quantities include physiological and pathological loading distributions, activation threshold, transition width, response kinetics, hysteresis, fatigue under repeated loading, threshold drift during storage or degradation, and susceptibility to off-target activation. These mechanics-specific variables provide the organizing basis for the translational challenges discussed below.

### 8.1. Mechanical Heterogeneity and Therapeutic Selectivity

#### 8.1.1. Defining a Clinically Acceptable Activation Window

Disease-associated mechanical values frequently overlap with the range observed in healthy tissues. Consequently, a mechanically responsive therapeutic cannot be designed around a single nominal disease value. Instead, design should be based on probability distributions describing mechanical exposure in both target and non-target tissues. If these distributions overlap substantially, a sharply defined activation threshold may still generate false-positive activation in healthy tissue or false-negative activation in diseased tissue.

A clinically useful activation window should therefore be evaluated in terms of mechanical sensitivity and specificity. The relevant engineering quantities include the lower and upper distributions of physiological exposure, the distribution associated with disease, the carrier activation threshold, and the width of the force–response transition. The therapeutic threshold should ideally lie within a region that maximizes activation in the diseased state while maintaining an adequate safety margin from normal physiological loading.

#### 8.1.2. Physiological Fluctuations and Off-Target Activation

Mechanical conditions are dynamic even in healthy individuals. Exercise, changes in systemic blood pressure, respiratory motion, cardiac cycling, posture, local vasoconstriction, and transient inflammatory responses can alter shear stress, pressure, strain, or tissue deformation. A carrier calibrated only against resting physiological values could therefore undergo unintended activation during normal physiological excursions.

Mechanically responsive systems should consequently be challenged across the full expected physiological loading envelope. Testing should include transient suprathreshold events, repeated subthreshold loading, loading-rate dependence, cyclic fatigue, recovery behavior, and cumulative structural damage. These measurements are particularly important for systems activated through carrier rupture, disassembly, deformation, or mechanically induced permeability changes.

#### 8.1.3. Spatial and Patient-to-Patient Heterogeneity

Mechanical heterogeneity also exists within individual lesions and across patients. Tumors can contain spatially distinct regions of matrix stiffness, solid stress, vascular compression, and interstitial pressure, while vascular lesions can exhibit strongly localized variations in shear stress and flow separation. A single therapeutic threshold may therefore produce nonuniform activation within one lesion and inconsistent behavior across patients.

Translation will require activation criteria that remain robust across this variability or a patient-stratification strategy in which the relevant mechanical parameter is measured before treatment. The latter approach is only practical when the mechanical biomarker can be measured noninvasively or with clinically acceptable procedures and when the measurement is sufficiently reproducible to guide therapeutic selection.

#### 8.1.4. Dual Mechanical–Biochemical Gating

Where healthy and diseased mechanical distributions overlap, mechanical activation alone may be insufficient to achieve therapeutic specificity. A dual-gating strategy could require both a mechanical condition and a biochemical condition before activation. Examples include elevated shear combined with binding to an inflammatory endothelial marker, stiffness-dependent material response combined with tumor-receptor recognition, or pressure sensitivity combined with a disease-associated enzymatic trigger.

Such an AND-gated architecture could reduce off-target activation by requiring independent physical and molecular evidence of disease. This approach also clarifies the role of mechanics within precision nanomedicine: mechanical information should complement rather than replace molecular targeting when either modality alone lacks sufficient specificity.

### 8.2. Measurement and Standardization of Mechanical Biomarkers

The success of mechanically responsive therapeutics depends on the ability to accurately characterize mechanical environments within living tissues. Despite significant technological advances, direct measurement of forces in vivo remains challenging.

Many biologically relevant mechanical variables are difficult to measure directly, including cellular traction forces, local matrix stresses, interstitial pressure gradients, nanoscale force distributions, and dynamic tissue strain. These quantities frequently vary over micrometer length scales and millisecond time scales, making measurement particularly challenging.

Several approaches have emerged for assessing tissue mechanics such as magnetic resonance elastography, ultrasound elastography, optical coherence elastography, atomic force microscopy, traction force microscopy, computational fluid dynamics, and finite-element modeling. While these methods provide valuable insights, each possesses important limitations related to resolution, invasiveness, accessibility, or clinical practicality.

For mechanics-guided therapeutics to become clinically viable, mechanical biomarkers must become as standardized and interpretable as molecular biomarkers. This requires validated measurements of stiffness, shear, pressure, strain, and deformation that are reproducible across imaging platforms, disease types, and clinical centers. For mechanically activated therapeutics, these measurements must ultimately be linked to the actual force or material state experienced by the carrier rather than only to an organ-level surrogate.

### 8.3. Multiscale Modeling and Mechanobiological Complexity

Mechanical forces operate across multiple spatial and temporal scales. A major challenge in mechanotherapeutics is understanding how nanoscale forces experienced by therapeutic materials translate into tissue-level biological outcomes.

Mechanobiological responses span nanometer-scale protein conformational changes, micrometer-scale cellular remodeling, millimeter-scale tissue transport, and organ-level disease progression. These processes also occur across time scales ranging from millisecond ion-channel activation to long-term tissue remodeling. Predictive models must therefore couple mechanics, transport, signaling, pharmacokinetics, pharmacodynamics, and disease evolution across multiple scales.

Current computational models often focus on isolated aspects of mechanobiology rather than comprehensive system-level integration. Future predictive frameworks must couple mechanics, transport phenomena, cellular signaling, tissue remodeling, pharmacokinetics, and pharmacodynamics. Developing such multiscale models remains a formidable challenge. A model becomes therapeutically useful only when it predicts a measurable carrier-level quantity, such as local force exposure, transport probability, activation fraction, tissue penetration, or therapeutic response, and is validated against experimental data.

### 8.4. Manufacturing Reproducibility and Mechanical Quality Attributes

For mechanically responsive therapeutics, manufacturing reproducibility must include preservation of the force–response behavior itself. Two batches with equivalent chemical composition and drug loading may not be functionally equivalent if differences in particle aggregation, geometry, crosslink density, supramolecular packing, polymer architecture, or interfacial adhesion alter the activation threshold.

The mechanical response should therefore be treated as a critical quality attribute. Batch-release testing may require measurement of activation threshold, force–response transition width, fraction of carriers activated at a defined load, activation kinetics, hysteresis, fatigue under repeated loading, and threshold drift during shelf storage. For shear-activated systems, standardized flow geometries and shear protocols would be required; for deformable or pressure-responsive systems, controlled loading and unloading protocols would be necessary.

Scale-up introduces an additional challenge because manufacturing processes can alter precisely the structural features responsible for mechanical sensitivity. A clinically translatable system must therefore demonstrate that its mechanical response is preserved across manufacturing scale, sterilization, storage, transport, and final administration.

### 8.5. Preclinical Validation and Evidence Required for Translation

Most directly mechanics-responsive nanotherapeutic systems remain preclinical; consequently, the field does not yet provide a large set of failed late-stage clinical programs specific to mechanical activation. The more immediate translational bottleneck is progression from proof-of-concept mechanical response to reproducible performance under realistic human mechanical conditions.

Preclinical models must reproduce the mechanical stimulus that the therapeutic is intended to exploit. Animal differences in vessel diameter, blood-flow rate, pressure, organ size, extracellular-matrix composition, and tissue deformation can shift the force experienced by a carrier relative to the corresponding human condition. Demonstration of efficacy in an animal model is therefore insufficient unless the relevant mechanical exposure is measured or modeled and shown to overlap the intended human activation regime.

A mechanics-responsive platform should also demonstrate an advantage that justifies its additional complexity. Appropriate comparisons include otherwise identical nonresponsive carriers, carriers with deliberately shifted activation thresholds, and conventional molecularly targeted formulations. Relevant endpoints include localization, therapeutic index, off-target activation, systemic toxicity, penetration, temporal control, and robustness across mechanical heterogeneity.

### 8.6. Regulatory Considerations for Force-Responsive Therapeutics

Mechanically responsive therapeutics introduce regulatory questions because the force–response characteristic becomes part of product performance. In addition to conventional requirements for identity, purity, stability, dose, and toxicity, regulatory evaluation may require standardized assays defining the mechanical activation threshold, response kinetics, off-target activation, durability under repeated loading, and reproducibility across manufacturing batches.

The appropriate regulatory pathway may also depend on whether the therapeutic functions primarily as a drug, biomaterial, device, or combination product. Long-term safety assessment should consider whether physiological changes over time alter activation behavior and whether repeated mechanical loading produces fatigue, irreversible structural change, or unintended release.

## 9. Testable Future Directions for Mechanics-Guided Nanomedicine

The future relevance of mechanics-guided nanomedicine should be evaluated through testable milestones rather than broad predictions. Three directions are proposed below with approximate time horizons, supporting evidence that would strengthen each proposition, and observations that would argue against it. These time horizons are intended as research benchmarks rather than forecasts of clinical adoption.

### 9.1. Near-Term Direction: Standardized Mechanical Characterization (3–5 Years)

A near-term milestone would be routine quantitative characterization of relevant mechanical environments in studies of nanotherapeutic transport and activation. Evidence supporting this development would include standardized reporting of shear stress, stiffness, strain, pressure, and viscoelasticity; interlaboratory validation of mechanical measurements; and demonstration that these variables predict therapeutic performance independently of conventional physicochemical parameters. This proposition would be weakened if mechanical measurements remain poorly reproducible across platforms or if inclusion of mechanical variables fails to improve prediction of transport, activation, or therapeutic response.

### 9.2. Mid-Term Direction: Validated Mechanics-Responsive Therapeutic Systems (5–10 Years)

A mid-term milestone would be independent validation of mechanics-responsive therapeutic systems across multiple preclinical models and physiologically realistic loading conditions. Supporting evidence would include reproducible activation windows, superiority over matched nonresponsive controls, quantitative correlation between local mechanical exposure and therapeutic activation, acceptable safety under physiological loading extremes, and successful reproduction by independent laboratories. This proposition would be weakened if activation thresholds prove too variable across patients or biological conditions, if normal physiological fluctuations produce substantial off-target activation, or if mechanically responsive systems fail to improve therapeutic index relative to simpler molecularly targeted approaches.

### 9.3. Longer-Term Direction: Patient-Specific Mechanics-Guided Therapeutic Design (>10 Years)

A longer-term milestone would be prospective use of patient-specific biomechanical measurements to guide nanotherapeutic selection, formulation, or dosing. Supporting evidence would require validated workflows linking imaging or biomechanical measurements to therapeutic design, computational models that predict carrier behavior under patient-specific conditions, and prospective demonstration that mechanics-informed selection improves therapeutic response or reduces toxicity beyond approaches based on molecular and anatomical information alone. This proposition would be weakened if patient-specific mechanical measurements add little predictive value, cannot be measured with sufficient reproducibility, or cannot be translated into actionable carrier-design or treatment decisions.

## 10. Conclusions

Mechanics influences nanotherapeutic performance at multiple levels, from vascular transport and tissue penetration to membrane interaction, intracellular trafficking, material activation, and mechanotransduction. The central proposition of this Perspective is that these effects should not be grouped into a single broad concept of “mechanical responsiveness.” Instead, mechanics should be distinguished according to its role as a transport determinant, an activation signal, or a biological therapeutic target.

The existing evidence is also heterogeneous. Shear-activated vascular systems and nanoneedle-mediated intracellular delivery provide direct examples in which mechanical interactions are deliberately engineered into therapeutic delivery, whereas several stiffness-, strain-, pressure-, adaptive-, and patient-specific strategies remain earlier-stage or prospective. Clear separation between these evidence levels is essential for realistic assessment of the field.

Progress toward clinical translation will require quantitative mechanical biomarkers, experimentally defined activation windows, evaluation under physiological fluctuations, force-specific quality-control criteria, and validation across mechanically heterogeneous patient populations. Where mechanical and healthy-tissue distributions overlap, combined mechanical and biochemical gating may provide greater specificity than either approach alone. In this form, interfacial mechanics becomes not a replacement for molecular nanomedicine, but an additional engineering dimension for designing more predictive and context-responsive therapeutic systems.

## Figures and Tables

**Table 1:** Mechanical-response strategies relevant to mechanics-guided nanotherapeutics. The evidence level is intentionally distinguished across categories because mechanically responsive nanotherapeutics do not yet constitute a uniformly mature therapeutic class.

Mechanical Variable	Representative Strategy	Physical Mechanism	Representative Application	Evidence Level	Translational Status
Shear stress	Shear-activated particle aggregates	Hydrodynamic stress exceeds interparticle cohesion, causing fragmentation and nanoscale cargo deployment	Thrombotic/stenotic vascular obstruction	Direct experimental demonstration	Preclinical
ECM stiffness	Mechanically dynamic hydrogels and stiffness-informed materials	Matrix mechanics alter deformation, permeability, ligand exposure, degradation, or cell–material interaction	Cancer, fibrosis	Indirect/early experimental evidence	Experimental
Cyclic strain	Stretch-sensitive polymers, dynamic hydrogels, mechanically adaptive scaffolds	Repeated deformation alters material structure or release behavior	Cardiac and musculoskeletal applications	Early experimental evidence	Experimental/preclinical
Pressure/compression	Deformable or pressure-sensitive materials	Compression or pressure alters carrier structure, permeability, or release	Tumor and pressure-associated disease	Limited direct evidence	Early experimental/conceptual
Nano–cell contact force	Nanoneedle interfaces	Local nanoscale membrane deformation or transient nanoperforation	Intracellular molecular and gene delivery	Direct experimental demonstration	Preclinical
Mechanical + biochemical dual gating	Multimodal responsive carrier	Mechanical activation permitted only when a second molecular condition is satisfied	Mechanically heterogeneous disease	Emerging concept	Conceptual/early experimental

## Data Availability

No new data were created or analyzed in this study. Data sharing is not applicable to this article.
